# Detection of Sialic Acid-Utilising Bacteria in a Caecal Community Batch Culture Using RNA-Based Stable Isotope Probing

**DOI:** 10.3390/nu7042109

**Published:** 2015-03-25

**Authors:** Wayne Young, Markus Egert, Shalome A. Bassett, Rodrigo Bibiloni

**Affiliations:** 1 AgResearch Ltd, Food Nutrition & Health Team, Food & Bio-based Products Group, Grasslands Research Centre, Private Bag 11008, Palmerston North 4442, New Zealand; E-Mails: shalome.bassett@agresearch.co.nz (S.A.B.); Rodrigo.Bibiloni@arlafoods.com (R.B.); 2 Microbiology & Hygiene Group, Faculty of Medical and Life Sciences, Furtwangen University, 78054 Villingen-Schwenningen, Germany; E-Mail: Markus.Egert@hs-furtwangen.de; 3 Arla Strategic Innovation Centre, Roerdrumvej 2, 8220 Brabrand, Denmark

**Keywords:** Sialic acid, piglet, microbiota, RNA-SIP, *Prevotella*

## Abstract

Sialic acids are monosaccharides typically found on cell surfaces and attached to soluble proteins, or as essential components of ganglioside structures that play a critical role in brain development and neural transmission. Human milk also contains sialic acid conjugated to oligosaccharides, glycolipids, and glycoproteins. These nutrients can reach the large bowel where they may be metabolised by the microbiota. However, little is known about the members of the microbiota involved in this function. To identify intestinal bacteria that utilise sialic acid within a complex intestinal community, we cultured the caecal microbiota from piglets in the presence of ^13^C-labelled sialic acid. Using RNA-based stable isotope probing, we identified bacteria that consumed ^13^C-sialic acid by fractionating total RNA in isopycnic buoyant density gradients followed by 16S rRNA gene analysis. Addition of sialic acid caused significant microbial community changes. A relative rise in *Prevotella* and *Lactobacillus* species was accompanied by a corresponding reduction in the genera *Escherichia*/*Shigella*, *Ruminococcus* and *Eubacterium*. Inspection of isotopically labelled RNA sequences suggests that the labelled sialic acid was consumed by a wide range of bacteria. However, species affiliated with the genus *Prevotella* were clearly identified as the most prolific users, as solely their RNA showed significantly higher relative shares among the most labelled RNA species. Given the relevance of sialic acid in nutrition, this study contributes to a better understanding of their microbial transformation in the intestinal tract with potential implications for human health.

## 1. Introduction

Sialic acids are a family of *N*-acylated neuraminic acids that have an important role in brain development, neuronal transmission and synaptogenesis [1]. The highest concentrations of sialic acids in the body are found in the brain, where they form an essential part of ganglioside structures [2], which steadily increase in concentration during infancy [3]. The rapid growth and development of the infant nervous system places substantial demands on the supply of nutrients, including sialic acid. Animal studies have shown a link between improved learning ability and levels of sialic acid in brain gangliosides and glycoproteins [4,5]. Human milk contains high concentrations of sialic acid attached to the terminal end of oligosaccharides, glycolipids and glycoproteins. Milk oligosaccharides, the largest source of sialic acid in human milk [6], are resistant to mammalian digestive enzymes and are therefore available for degradation by the large bowel microbiota [7]. Once they reach the large bowel, milk oligosaccharides can modulate immunity by stimulation of favourable microbes such as *Lactobacillus* and *Bifidobacterium* [8], and by inhibiting pathogen binding [9]. Sialic acid-containing gangliosides are also an important component of milk. Infant formula enriched with GD3, the most abundant ganglioside in human colostrum, has been shown to increase cognitive development in infants [10]. While the majority of ingested gangliosides are likely to be absorbed in the small intestine, measurable quantities are able to reach the large bowel and influence the composition and activity of the resident microbiota there [11,12,13].

Stable isotope probing (SIP) was introduced more than 15 years ago to identify microbes that use a particular growth substrate under *in situ* conditions [14,15]. This strategy relies on the incorporation of a substrate that is highly enriched in a stable isotope (e.g., ^13^C) by metabolically active microorganisms in an environmental sample. By the selective recovery and analysis of their isotope-enriched DNA or RNA, it becomes possible to identify such microorganisms. Initially used mainly in the field of soil and water microbiology [16,17], the SIP technique has also more recently been used to study digestive processes involving simple and more complex sugars [18,19,20].

Although intestinal bacteria have been shown to produce glycosidases capable of desialylating milk gangliosides [21], little is known about the fate of ingested sialic acid and sialic acid-containing nutrients in the intestinal tract. The aim of our study was to identify bacteria that utilise sialic acid in a complex intestinal community. We chose the piglet model because it represents a more physiologically relevant animal model for studying human health than the more commonly used rodent models [22]. We used the established RNA-stable isotope probing approach and ^13^C-labelled sialic acid as a model substrate. To the best of our knowledge, our study is the first that addresses the effect of sialic acid on a complex intestinal community and its fate regarding assimilation by intestinal microorganisms.

## 2. Experimental Section

### 2.1. Animal Information

Eight healthy male Large Pure White Cross 21 day old piglets, from the same litter, purchased from a commercial farm in Wanganui, New Zealand were used in this study. During the acclimatisation period, animals were housed in groups of two or three in piglet pens for four days and provided creep feed and drinking water *ad* *libitum*. After four days, the piglets were anaesthetised with Zoletil 100 (125 mg/mL Tiletamine HCI, 125 mg/mL Zolezepam HCL) reconstituted with Ketamine and Xylazine at a final solution concentration 50 mg/mL of each drug and administered at a dose rate of 0.3 mL of the mixed solution/10 kg body weight. Once a sufficient level of sedation was reached, the piglets were euthanised by an intra-cardiac injection of sodium pentobarbitone (300 mg/mL) at 120 mg/kg body weight to collect intestinal contents. Caecal content samples were immediately placed into tubes that were previously flushed with CO_2_. All animal procedures were approved by the Grasslands Animal Ethics Committee, Palmerston North, New Zealand (tissue collection application number 118) operating under the New Zealand Animal Welfare Act 1999.

### 2.2. Caecal Cultures

Fresh piglet caecal contents were cultured at a 100-fold dilution in basal medium (8.5 g NaCl, 0.3 g KH_2_PO_4_, 0.6 g Na_2_HPO_4_, 0.24 g MgSO_4_, 0.011 g CaCl_2_, 4 g NH_4_Cl, 1 mg resazurin, and 0.25 mg cysteine-HCl per litre, pH 7.1) in gas-tight Hungate tubes at 37 °C in a total volume of 5 mL under anaerobic conditions for 24 h. Prior to use, the basal mineral medium was boiled and flushed with a mixture of CO_2_ and N_2_ in equal parts prior to autoclave sterilisation. To characterise the changes in the caecal community induced by culture conditions, caecal contents from five piglets were cultured individually with basal medium alone (control cultures). To identify sialic acid-utilising members of the intestinal microbiota, caecal contents from three piglets were cultured independently with basal medium alone, or basal medium supplemented with ^13^C-sialic acid (*N*-acetyl-d-[1,2,3-^13^C_3_]neuraminic acid, molecular weight 312.24; Omicron Biochemicals, IN, USA), at a final concentration of 2 mg/mL for 24 h, which is a similar level to that found in faeces of breast-fed infants [23].

### 2.3. Nucleic Acid Extraction

Bacterial cells from all cultures were pelleted for nucleic acid extraction by centrifugation at 5000 × *g* for 10 min at 4 °C. To characterise the effects of culture conditions on the piglet caecal composition, DNA was extracted from piglet caecal contents (*n =* 5) and corresponding caecal content control cultures in basal medium using a NucleoSpin Soil kit following the manufacturer’s instructions (Macherey Nagel, Düren, Germany). To analyse caecal microbial utilisation of sialic acid, RNA was extracted from caecal communities cultured in basal medium with ^13^C-sialic acid and corresponding control cultures in basal medium alone (*n =* 3) using a previously described phenol/chloroform bead beating method [24], with the following modifications. Briefly, RNA pellets were dissolved in 50 μL of nuclease-free water and residual DNA was removed by incubating the RNA for 1 h at 37 °C in a solution containing 20 μL of 5X DNase I buffer (30 mM MgCl_2_, 10 mM NaCl, 200 mM Tris-HCl, pH 7.9), 1.5 μL of RNase-free DNase I (Roche, Basel, Switzerland) at a stock concentration of 10 U/μL, and 28.5 μL of nuclease-free water. Samples were then further purified using Qiagen RNeasy (Qiagen, Venlo, Netherlands) spin columns according to the manufacturer’s instructions.

### 2.4. RNA-SIP

Because of the higher synthesis rate of RNA compared to DNA [25], RNA was analysed to determine which members of the microbiota were able to utilise sialic acid. Density gradient centrifugation of RNA from ^13^C-sialic acid containing cultures and the corresponding control cultures without sialic acid were performed using a previously described method [18] with the following modifications: the centrifugation solution was prepared by mixing 5.4 mL CsTFA stock solution with a buoyant density of 2 g/mL (Amersham, GE Healthcare, Little Chalfont, UK), 1.2 mL of gradient buffer (0.1 M Tris-HCl, pH 8.0, 0.1 M KCl, 1 mM EDTA) containing 600 ng of RNA, and 210 μL of formamide into 6 mL crimp top ultracentrifugation tubes (Sorvall, Waltham, MA, USA). Prior to centrifugation, the density of each prepared gradient was checked with an AR200 refractometer (Reichert, Buffalo, NY, USA) and adjusted to similar buoyant densities (~1.775 g/mL) by adding CsTFA stock solution or gradient buffer, as required. Each gradient was then centrifuged at 130,000 × *g* for 65 h at 20 °C. After centrifugation, 15 equal fractions from bottom to top (~400 μL) were collected from each gradient by displacing the gradient solution from the top of the tube with water under a constant 400 μL/min flow rate. The refractory indices of each fraction from all gradients were determined using the refractometer and the density determined by weighing a 50 μL aliquot. RNA from each fraction was precipitated by adding one volume of isopropanol. Precipitated RNA was washed once with 150 μL of ice cold 70% ethanol and re-dissolved in 30 μL of TE buffer.

### 2.5. Reverse-Transcription and PCR

RNA from density gradient fractions was reverse-transcribed to cDNA using a SuperScript VILO cDNA Synthesis Kit (LifeTech, Carlsbad, CA, USA) and random primers following the manufacturer’s protocol. The relative amounts of bacterial 16S rRNA cDNA in each fraction was then measured by quantitative PCR (qPCR) with a Corbett Rotor-Gene 6000 using universal bacterial primers F_Bact 1369 (CGGTGAATACGTTCCCGG) and R_Prok1492 (TACGGCTACCTTGTTACGACTT) [26] with the following thermal cycling protocol: 95 °C for 3 min followed by 40 cycles of 95 °C, 60 °C for 30 s, and 72 °C for 30 s. The amplification mixture consisted of 10 μL KAPA SYBR^®^ FAST Universal 2X qPCR Master Mix (Kapa Biosystems, Wilmington, MA, USA), 1 μL of each primer (10 pmol/μL), 1 μL cDNA template, and 7 μL of nuclease-free water. Concentrations of cDNA were then calculated against a standard curve generated from serial dilutions of *Escherichia* *coli* DH5-α DNA and expressed as percentage cDNA content relative to the maximum amount of DNA found among the fractions for each gradient.

Identification of bacterial groups represented in DNA from fresh piglet caecal content, DNA from caecal cultures in basal mineral medium, and cDNA from density gradient fractions of caecal contents cultured with ^13^C-sialic acid was carried out by barcode tagged PCR amplification of the V456 region of the 16S rRNA gene. The primers and PCR conditions used were based on a previously published method [27] and summarised as follows: the primers used contained GS FLX adapter sequences, a unique 8 nucleotide ‘barcode’, and template specific sequences; forward primer (CGTATCGCCTCCCTCGCGCCATCAG NNNNNNNN AGGCCAGCAGCCGCGGTAA), and reverse primer (CTATGCGCCTTGCCAGCCCGCTCAG GCCRRCACGAGCTGACGAC), with ‘N’ indicating barcode nucleotides. The amplification mixture consisted of 50 μL of Roche FastStart PCR Master Mix (Roche, Basel, Switzerland), 2 μL of each primer (20 pmol/μL), 42 μL nuclease-free water, and 4 μL of cDNA or DNA template adjusted to a concentration of 50 ng/μL. Amplification reactions were carried out on MasterCycler ProS thermocycler (Eppendorf AG, Hamburg, Germany) using the following conditions 95 °C for 4 min, 25 cycles of (95 °C for 30 s; 49 °C for 30 s; 72 °C for 60 s) and 72 °C for 7 min. The PCR product size was 604 bp. Products were purified using Roche High Pure PCR Product Purification kits according to the manufacturer’s instructions. Purified products were pooled in equivalent quantities and sent to Macrogen (Seoul, Korea) for unidirectional sequencing from the forward primer using the Roche GS-FLX sequencer with Titanium chemistry.

### 2.6. Sequence Analysis

Sequences were processed using Qiime 1.8 [28]. Reads were quality filtered using default settings and assigned to corresponding samples according to their barcode sequence. The resulting de-multiplexed sequences were chimera checked using the USEARCH method against the Greengenes alignment (release GG_13_8). Chimeric sequences were removed from subsequent analyses. Sequences showing 97% or greater similarity were clustered into operational taxonomic units (OTUs) using the UCLUST method and representative sequences were assigned taxonomies using the RDP classifier. Faith’s Phylogenetic Diversity estimates were performed using the core_diversity_analyses.py script in Qiime 1.8.

### 2.7. Statistics

Statistical analyses were conducted using R 3.0.2 [29]. Differences in the proportions of bacterial taxa between ‘dense’ and ‘light’ fractions from ^13^C-sialic acid cultures and between ‘light’ fractions from control and ^13^C-sialic acid cultures were assessed using the Wilcoxon rank sum test. Hierarchical cluster analysis of bacterial profiles was performed using distances calculated from centered Pearson’s correlation and average linkage clustering.

## 3. Results

### 3.1. Characterisation of Fresh and Cultured Piglet Caecal Microbiota Composition

Pyrosequencing analysis of DNA extracted from freshly collected caecal communities and caecal contents cultured in basal medium generated a total of 169,193 reads with a maximum of 20,528 and a minimum of 9550 reads per sample.

**Figure 1:**
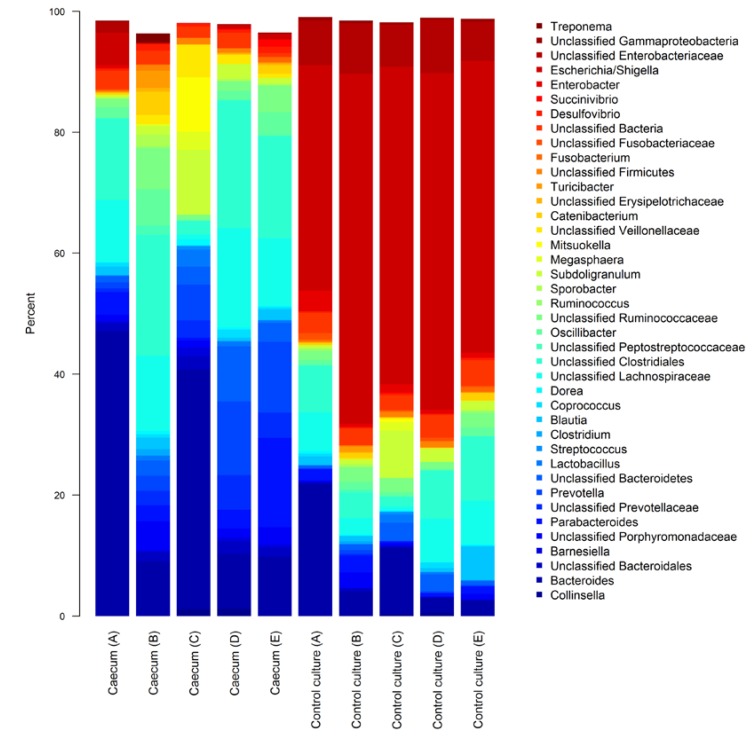
Stacked barplots showing genus level composition of fresh piglet caecal community (Caecum) and caecal contents cultured in basal mineral medium (Control culture). Letters in parentheses identify the piglet source for caecal contents. The bacteria shown are the 40 taxa with the highest mean relative abundance across all samples.

As expected, the majority of the microbial community from fresh caecal contents were assigned to the *Firmicutes* (46.5% ± 6.1% SEM; standard error of the mean) and *Bacteroidetes* (46.6% ± 5.6% SEM) phyla, with the *Proteobacteria* (3.5% ± 1.3% SEM) forming the most relatively abundant of the minor taxa. Culturing in basal medium led to a large shift in the caecal community composition (Figure 1), with *Proteobacteria* becoming the most relatively prevalent phylum (59.9% ± 3.0% SEM), of which the *Escherichia*/*Shigella* genera formed the largest group (47.8% ± 3.1% SEM). The rise in *Proteobacteria* from culture conditions corresponded with a drop in *Firmicutes* and *Bacteroidetes* proportions (22.2% ± 3.4% SEM and 13.2% ± 3.1% SEM, respectively).

### 3.2. Detection of Piglet Caecal Bacteria Able to Utilise ^13^C-sialic Acid

**Figure 2:**
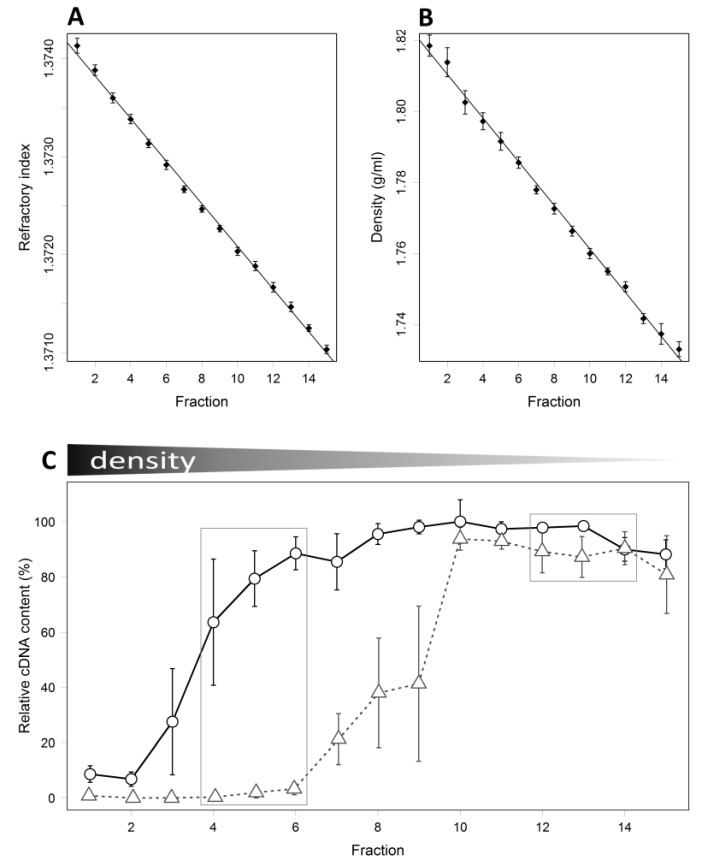
Refractory index (**A**) and buoyant density (**B**) curves from isopycnic buoyant density gradients. Means and standard errors (SEM) of six gradients are shown; (**C**) mean relative content of cDNA ± SEM in fractions collected from density gradients determined by qPCR in cultures containing ^13^C-sialic acid (circles, *n =* 3) or corresponding control cultures (triangles, *n =* 3). Boxes indicate fractions analysed for microbiota composition.

To identify piglet caecal bacteria able to utilise sialic acid, caecal contents were cultured with ^13^C labelled sialic acid followed by RNA extraction and isopycnic buoyant density gradient separation to detect incorporation of ^13^C into bacterial RNA. The buoyant density and refractory curves from density gradients (Figure 2A,B) showed that gradients of an appropriate density spectrum for separating different density RNA were obtained when compared to previous published RNA-SIP studies [20,30]. Quantitative PCR analysis showed bacterial RNA from cultures in basal medium alone was present in density gradient fractions 7 to 15, which had buoyant densities between 1.78 and 1.72 g/mL, respectively. In caecal cultures supplemented with ^13^C-sialic acid, RNA was present in density gradient fractions 4 to 15, with fractions 4–6 having buoyant densities between 1.78 and 1.80 g/mL (Figure 2C). Nucleic acids from bacteria with the highest rates of ^13^C-sialic acid assimilation are likely to be found in fractions 4 to 6 due to an increase in their density as a result of the higher incorporation of ^13^C in place of ^12^C compared to bacteria represented in fractions 7 to 15. For statistical analyses, the mean taxonomic composition of fractions 4–6 (‘dense’ fractions) and fractions 12–14 (‘light fractions’) from each culture were compared to each other (*n =* 3 independent cultures). Fractions 4–6 from control cultures did not yield any detectable PCR products.

**Figure 3:**
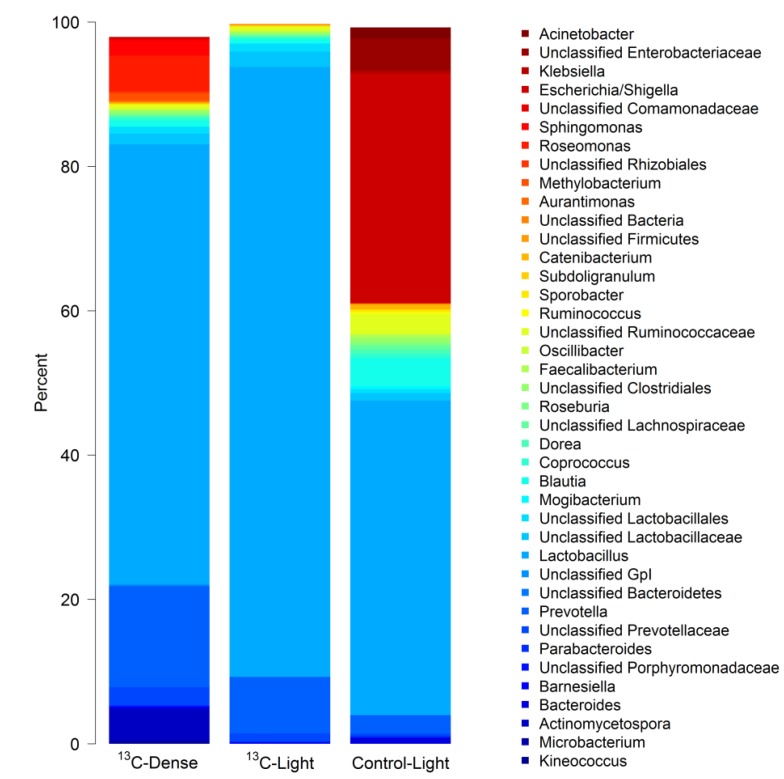
Stacked barplot showing the mean genus level composition of density gradient fractions from independent cultures (3 piglets). Bars labelled ^13^C-Dense represent the mean composition of fractions 4–6 in cultures with ^13^C-sialic acid; ^13^C-Light represents the mean composition of fractions 12–14 in cultures with ^13^C-sialic acid; Control-Light represents the mean composition of fractions 12–14 in control cultures without sialic acid. The bacteria shown are the 40 taxa with the highest mean relative abundance across all samples.

**Figure 4:**
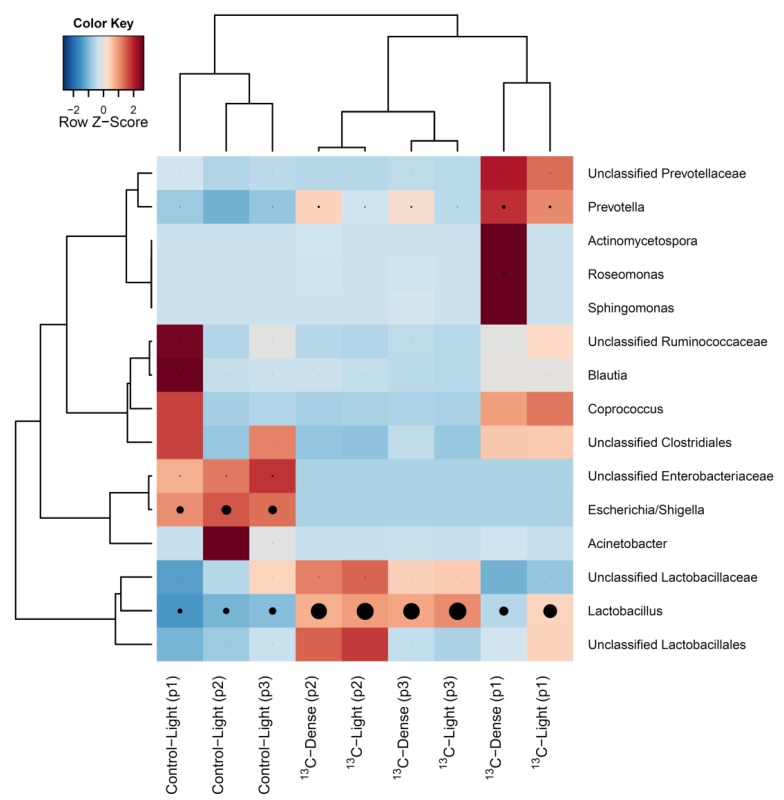
Heatmap showing hierarchical clustering of bacterial composition profiles of the 15 most relatively abundant taxa in cDNA from RNA-SIP density gradient fractions. Taxa are indicated by row labels and gradient fraction type indicated by column labels; ‘light’ fractions from control culture (Control-Light), ‘light’ fractions from ^13^C-sialic acid cultures (^13^C-Light), and ‘dense’ fractions from ^13^C-sialic acid cultures (^13^C-Dense). Identification in parentheses indicates the piglet source. Heatmap colour (blue to dark red) signifies the relative prevalence of each taxon across samples and black circles show absolute proportions for each taxa within a sample, with circle size proportional to sequence abundance.

Pyrosequencing analysis of RNA-SIP fractions generated a total of 130,566 reads with a maximum of 16,952 and a minimum of 1667 reads per sample. The minimum number of reads (1667) was the value used for calculating Faith’s phylogenetic diversity estimates and Unifrac phylogenetic distance analyses. Analysis of the bacterial composition in ‘dense’ fractions (fractions 4–6) of ^13^C-sialic acid-fed cultures revealed a complex community consisting of many bacterial taxa (Figure 3), which was equal in complexity to the bacterial diversity present in ‘light’ fractions (fractions 12–14) from both control and ^13^C-sialic acid-fed cultures (Faith’s Phylogenetic Diversity estimate ± standard deviation; ^13^C-dense = 22.9 ± 4.1; ^13^C-light = 21.1 ± 3.1; Control-light = 24.1 ± 3.0, *p* = 0.41). Although there was large inter-piglet variation, hierarchical clustering analysis showed bacterial profiles of density gradient fractions from cultures with ^13^C-sialic acid were clearly separated from those of control cultures (Figure 4).

**Figure 5:**
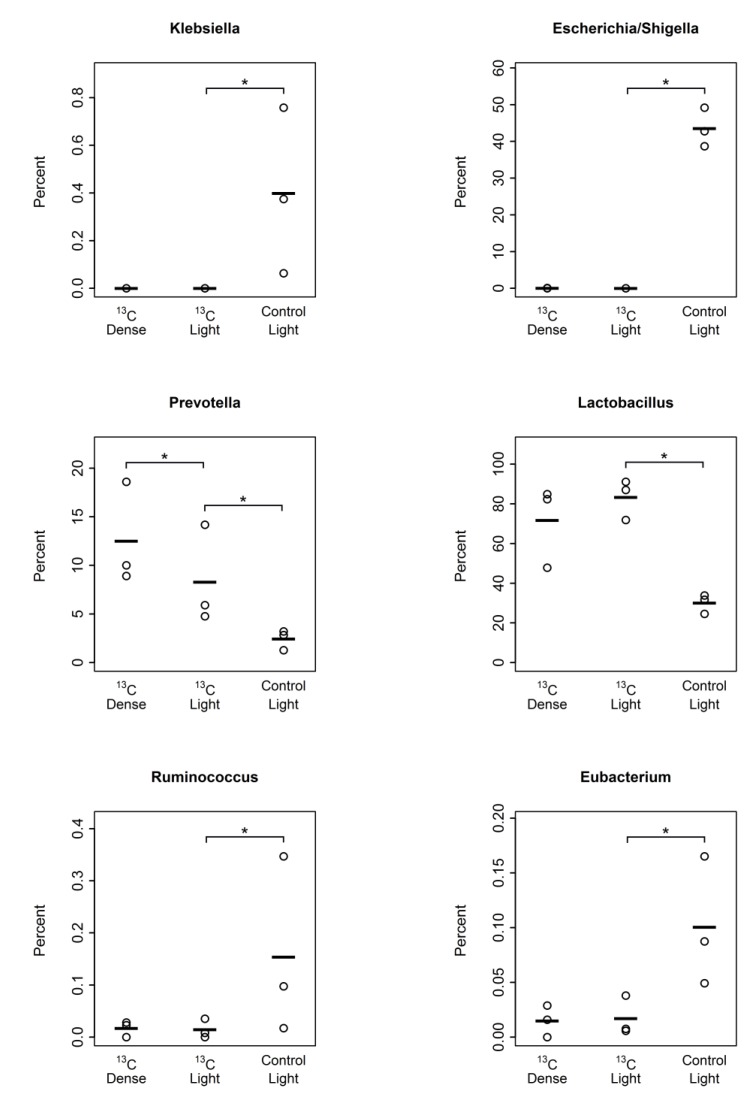
Plots of genus-level bacterial taxa significantly altered (*p <* 0.05) between fractions 12–14 in control cultures without sialic acid (Control Light, *n =* 3), fractions 12–14 in cultures with ^13^C-sialic acid (^13^C Light, *n =* 3), and fractions 4–6 in cultures with ^13^C-sialic acid (^13^C Dense, *n =* 3). Points indicate the percentage of total community and lines indicate the mean. * Indicates significant difference (*p <* 0.05) in relative proportions determined by Wilcoxon rank sum test.

The most prominent bacterial genera that showed significantly higher relative abundances (*p <* 0.05) in the presence of sialic acid included *Lactobacillus* (Control-Light 30.3% ± 2.8 SEM; ^13^C-Light 83.3% ± 5.8% SEM) and *Prevotella* (Control-Light 2.4% ± 0.6% SEM; ^13^C-Light 8.3% ± 2.9% SEM). *Prevotella* was the only genus showing a significantly greater proportion in the ‘dense’ fractions of ^13^C-sialic acid-fed cultures (^13^C-Dense 13.5% ± 2.5% SEM) (Figure 5). Conversely, proportions of *Eubacterium*, *Ruminococcus*, *Klebsiella*, and *Escherichia*/*Shigella* were significantly reduced (*p <* 0.05) in the ‘light’ fractions from ^13^C-sialic acid compared to the ‘light’ fractions from control cultures (Figure 5).

## 4. Discussion

In our pilot study presented here, simple batch incubations were used to study the effect of sialic acid on the structure and function of the caecal microbiota of piglets. In this model, culture conditions did appear to favour the *Proteobacteria* in the absence of an added carbon source, in comparison to the microbial composition of fresh caecal contents. Indeed, the lack of fermentable nutrients may explain the relatively higher prevalence of *Proteobacteria* at the expense of saccharolytic bacteria within *Firmicutes* and *Bacteroidetes* phyla. In our study, we used a nutrient-poor basal medium to maximise the incorporation of ^13^C into bacterial RNA. Despite these changes, the model communities still showed most major groups of bacteria present in the fresh caecal communities, and hence appeared suitable for further investigations. In particular, changes seen in cultured community composition after the addition of sialic acid showed that the bias towards *Proteobacteria* was at least partly reversible.

SIP technology relies on the premise that if an organism assimilates a substrate labelled with a stable or ‘heavy’ isotope, its cell constituents, including nucleic acids, become labelled with that isotope. The separation of ‘heavier’ labelled nucleic acids (‘dense’ fractions) from unlabelled nucleic acids (‘light’ fractions) achieved by density gradient centrifugation allows further identification of the microorganisms that were able to assimilate the labelled substrate. This approach makes it possible to unlock the ‘microbial black box’ and therefore link the consumption of a particular compound in the environment to the identity of the utiliser [31]. For example, RNA-SIP has been successfully applied to identify starch-utilising bacteria in an *in vitro* model of the human large bowel [19] and to show that *Bifidobacterium* *animalis* was the primary degrader of ^13^C-labelled inulin *in vivo* using a rat model [20]. In this study, we aimed to identify bacteria capable of utilising free sialic acid in a complex cultured piglet caecal community. Sialic acids, in particular *N*-acetylneuraminic acid, are common in nature and have important biological functions ranging from being an essential nutrient for brain development [32] to enabling pathogenic bacteria to evade host detection [33]. However, little is known about their fate in the large bowel ecosystem. Using ^13^C-probing and high throughput sequencing, we have shown that much of the intestinal microbiota in a piglet culture model may be capable of assimilating sialic acid based on the diversity of bacteria with ^13^C labelled RNA.

Our data also shows that some members of the microbial community are better able to utilise sialic acid than others. The proportion of *Prevotella* was markedly higher in the ‘dense’ fraction of ^13^C-sialic acid fed cultures, indicating that these members of the community were the most adept at utilising sialic acid, as demonstrated by the substantial incorporation of ^13^C into their ribosomal RNA. The genus *Lactobacillus* was also more relatively abundant in cultures containing ^13^C-sialic acid, compared to control cultures. Members of the genus *Prevotella*, and to a lesser extent, the genus *Lactobacillus*, are known for their ability to degrade and utilise mucin [34,35], a source of host-derived sialic acid. Therefore, it seems likely that members of genera *Prevotella* and *Lactobacillus* are most able to benefit from the presence of free sialic acid, even in a complex community where other bacteria may also be competing for this resource. Our results are also supported by a previous study showing sialylated human milk oligosaccharides can support the growth of *Lactobacillus* *delbrueckii*, whereas fucosylated oligosaccharides did not [8]. Furthermore, other members of the bacterial community are also known to desialylate macromolecules, which could contribute to the sialic acid pool [36]. Overall, the community profile of the ‘light’ fractions from ^13^C-sialic acid cultures were more similar to the ‘dense’ fractions from the same ^13^C-sialic acid culture, rather than to the ‘light’ fractions from the control cultures, as might be expected. However, because only three of the nine carbon atoms in the ^13^C-sialic acid used were the heavier ^13^C isotope, it is likely that bacteria represented in the ‘light’ fractions from ^13^C-sialic acid cultures were also sialic consumers, but had simply incorporated more ^12^C into their RNA than bacteria found in the ‘dense’ fraction. Furthermore, it is possible that some bacteria may become enriched by using sialic acid for energy production without assimilating its atoms into their RNA. In this way, they may benefit from the addition of ^13^C-sialic acid without becoming labelled. While it seems likely that bacteria enriched in the ‘dense’ fractions are the most proficient users of sialic acid, it cannot be ruled out that some of the bacteria in these fractions are gaining ^13^C through metabolism of by-products, such as acetate or lactate, released by primary sialic acid consumers. Additional experiments using different time points would be useful for quantifying rates of cross-feeding. Furthermore, because incorporation of ^13^C into RNA is likely to occur at differing rates with differing numbers of labelled RNA molecules per cell, ^13^C-RNA is likely to be distributed throughout the gradient, rather than in a discrete ‘band’. The caecal content itself will contain other sources of carbon originating from ingested food that could also be incorporated into RNA by bacteria stimulated to grow in the presence of sialic acid. Moreover, despite the fact that cultures systems do not in general take into account interactions with glycomic motifs at the lumen/host interface, the caecal contents investigated here may also have contained endogenous substrates of host origin, such as mucin, which could have been a source of unlabelled sialic acid.

Members of the genera *Eubacterium* and *Ruminococcus* were reduced in the presence of sialic acid, indicating that they were less able to utilise sialic acid, or that they were being out-competed for this resource by other members of the community. Indeed, it has been shown that in a mixed microbial community, *Eubacterium* *rectale* preferentially utilise complex plant-based polysaccharides over simple mono-saccharides [37]. While some *Ruminococcus* species are able to grow on sialylated substrates [38], it is likely that they also have a preference for complex polysaccharides, as shown by the extensive fibrolytic enzyme systems present in their genomes [39,40]. The proportions of the *Escherichia*/*Shigella* group were also substantially reduced in the presence of sialic acid, which at first would seem counter-intuitive as *E. coli* is known to grow on sialic acid [33,41]. However, it is possible that this reduction in proportions was a reflection of the relative increase in *Prevotella* and *Lactobacillus*, rather than a decrease in absolute numbers of *Escherichia*/*Shigella*. Indeed, it has been suggested that the ability of commensal bacteria to out-compete other bacteria for sialic acid may be critical for preventing infection by pathogens [42]. Colonisation of germ-free mice by *Bacteroides* *thetaiotamicron* results in the liberation of sialic acid from mucin, which, in the absence of a normal microbiota, can lead to the growth of the pathogens *Salmonella* *typhimurium* and *Clostridium* *difficile* [42]. Interestingly, it has also been shown that fimbriae expression in *E. coli* K12 is suppressed in the presence of excess sialic acid [43], which may be an adaptive response to activation of the host immune defence system. A reduction in fimbriae expression is likely to lead to reduced adherence, which in turn may lead to increased wash-out *in vivo* and therefore a decrease in *E. coli* abundance. This idea is supported by a study showing that infant formula fortified with gangliosides reduced faecal levels of *E. coli* in pre-term new-born infants [11].

Our data suggest that much of the large bowel microbiota can potentially utilise free sialic acid. However, sialic acids are also found conjugated to a variety of nutrients such as gangliosides and oligosaccharides. While the identity of large bowel bacteria capable of breaking down these substrates have yet to be fully characterised, studies suggest the ability to utilise milk oligosaccharides are restricted to a small number of species, most of which appear to be bifidobacteria [7,8,44]. Similarly, some bifidobacteria, in particular *B. infantis* and *B. bifidum*, have also been shown to possess the ability to degrade the milk gangliosides GM3 and GD3 [45]. Our results show that even if the ability to break down complex glycolipids, glycoproteins, and oligosaccharides is not universal among the intestinal community, any breakdown products that are released will be readily utilised by other bacteria present. Further studies using different concentrations of labelled substrate and time-points may provide additional details of the trophic interactions involved in bacterial utilisation of sialic acid.

## 5. Conclusions

In conclusion, we have demonstrated that sialic acid is readily consumed by many members of the intestinal microbiota, but with differing proclivities. This difference in ability to metabolise sialic acid may have important consequences for the host as shown by the growth of bacteria such as *Lactobacillus*, and the reduction in potential pathogens such as *Escherichia*/*Shigella*, and *Klebsiella*. Our study provides novel insights into the role of the intestinal microbial community in the metabolism of sialic acid, a critical nutrient for brain development and function. Furthermore, this study also highlights the importance of RNA-SIP technology for linking community composition to function.
